# Clinical Trial Findings and Drug Development Challenges for Curcumin in Infectious Disease Prevention and Treatment

**DOI:** 10.3390/life14091138

**Published:** 2024-09-09

**Authors:** Mohamed El Oirdi, Mohd Farhan

**Affiliations:** 1 Department of Life Sciences, College of Science, King Faisal University, Al Ahsa 31982, Saudi Arabia; 2 Department of Basic Sciences, Preparatory Year, King Faisal University, Al Ahsa 31982, Saudi Arabia; mfarhan@kfu.edu.sa; 3 Department of Chemistry, College of Sciences, King Faisal University, Al Ahsa 31982, Saudi Arabia

**Keywords:** curcumin, antimicrobial activity, clinical trials, therapeutic uses

## Abstract

Since ancient times, turmeric, scientifically known as *Curcuma longa*, has been renowned for its therapeutic properties. Recently, extensive documentation has highlighted the prevalence of microbial diseases without effective treatments, the increased expense of certain antimicrobial medications, and the growing occurrence of antimicrobial drug resistance. Experts predict that drug resistance will emerge as a significant global cause of death by the middle of this century, thereby necessitating intervention. Curcumin, a major curcuminoid molecule, has shown extensive antimicrobial action. Improving and altering the use of natural antimicrobial agents is the most effective approach to addressing issues of targeted specificity and drug resistance in chemically synthesized medicines. Further research is required to explore the efficacy of curcumin and other natural antimicrobial substances in combating microbial infections. The solubility and bioavailability of curcumin impede its antimicrobial capability. To enhance curcumin’s antimicrobial effectiveness, researchers have recently employed several methods, including the development of curcumin-based nanoformulations. This review seeks to compile the latest available literature to assess the advantages of curcumin as a natural antimicrobial agent (particularly antiviral and antibacterial) and strategies to enhance its medical efficacy. The future application of curcumin will help to alleviate microbial infections, thereby promoting the sustainability of the world’s population.

## 1. Introduction

*Curcuma longa*, a historical substance, has been employed in various capacities such as seasoning, a preservative, a dye, and within the realm of traditional medicinal practices. Curcumin represents one of the three predominant pigmented constituents (inclusive of demethoxy and bisdemethoxy derivatives) derived from the rhizomes of *Curcuma longa*, a plant cultivated predominantly in Asian regions [1]. The substance is frequently known by various names, such as turmeric, curcumin, Indian saffron, and haldi [2]. In the context of the COVID-19 pandemic, a significant surge in research investigations has been noted in the previous couple of years regarding methodologies for obtaining curcumin and elucidating its bioactive characteristics [2,3,4,5,6,7]. The worldwide curcumin market reached a valuation of approximately USD 55 million by the conclusion of 2017 [2]. The expansion of its application sectors is anticipated to lead to a projected increase in the size of the global curcumin market to USD 155 million by the year 2027 [2]. These projections stem from an analysis of the escalating number of publications focusing on the antioxidant, anti-inflammatory, and anti-cancer attributes, alongside the increasing availability and accessibility of Ayurvedic medicinal products, cosmetics, food supplements, and natural additives in the consumer markets of developed nations [1,2,3,5,7,8,9,10,11]. Leading the market for food, nutritional supplements, and cosmetics containing curcumin is North America, closely followed by Europe, with these products achieving their highest sales in 2019. India stands out as the primary producer of curcumin extracted from turmeric, contributing to more than 80% of the global production [2].

**Figure 1:**
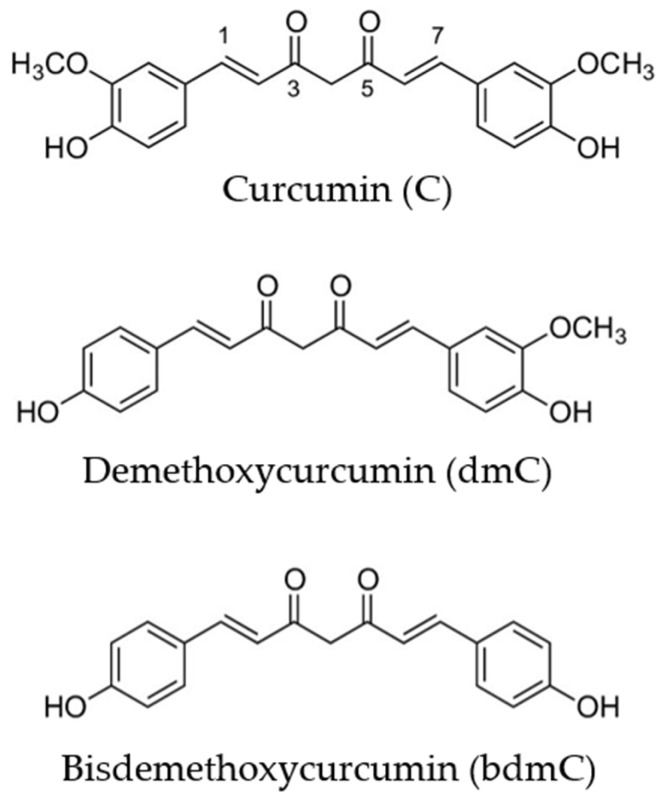
Chemical structure of curcumin, demethoxycurcumin, and bisdemethoxycurcumin.

Nevertheless, the forecasts provided are impacted by a considerable level of uncertainty as a result of operational difficulties associated with the acquisition of primary resources [2] and the distribution of completed goods in the consumer market, which have been impacted by the COVID-19 pandemic [8]. Curcumin is widely employed as a phytochemical in research on many diseases [7,9], with a specific focus on its antioxidant [10] and anti-inflammatory effects [11], particularly in the context of cancer treatment [1,12]. The present article underscores the recently uncovered domains where antiviral and antibacterial capabilities could potentially be harnessed. Methods for improving the aqueous solubility of curcumin via various treatments, along with the influence or synergistic effects of the components integrated into delivery systems, have been investigated. This research introduces innovative approaches for enhancing the stability and solubility attributes of curcumin, which bear considerable significance in medical and nutritional contexts. Moreover, this discourse will examine the advantages and imperative nature of employing natural compounds in the realm of promoting and advancing human health. The molecular structures of the primary curcuminoids are depicted in Figure 1.

This study synthesized all accessible data concerning the antimicrobial attributes of curcumin, alongside advancements in nanotechnology, and clinical trials aimed at enhancing curcumin’s bioavailability. The most recent iterations of databases such as Google Scholar, Scopus, Web of Science, and Pubmed were employed to identify relevant studies. Terms particularly such as “*Curcuma longa*”, “turmeric”, “curcumin”, “curcuminoids”, “infections”, “antimicrobial activities”, and “nanoparticles” were utilized as keywords during the online search.

## 2. Bioavailability and Metabolism of Curcumin

Due to a rapid hepatic metabolism, swift systemic clearance, and inadequate absorption in the small intestine, the bioavailability of curcumin in humans is notably low when administered at a dosage of 12 g/day [13]. Following absorption, a minor fraction of curcumin undergoes metabolic transformation, while the bulk of it is excreted without undergoing any metabolic alterations. The metabolic transformation of curcumin occurs through a two-phase process, with the initial reduction step being carried out by enterocytes and hepatocytes in the presence of reductases. Among the reduction byproducts, octahydrocurcumin (hexahydrocurcuminol) stands out, alongside dihydrocurcumin, tetrahydrocurcumin, and hexahydrocurcumin [14]. An unidentified enzyme along with alcohol dehydrogenase and nicotinamide adenine dinucleotide phosphate (NADPH)-dependent reductase catalyze the reduction reaction of curcumin [15]. The enzyme responsible for curcumin degradation was isolated [16]. andthis research concluded that, a purified enzyme was found to undergo a two-step reduction process in the microbial metabolism of curcumin. During this process, curcumin is transformed into dihydrocurcumin, an intermediate product, and finally into tetrahydrocurcumin, the ultimate product, with NADPH playing a crucial role [16]. In both living organisms and laboratory settings, glucuronic acid and sulfate readily conjugate with curcumin and its metabolites. When glucuronyl transferase and sulfotransferase enzymes are present, glucuronidation and sulfation reactions can occur. The intestines and liver of rats and humans are responsible for the glucuronidation and sulfation of curcumin [14]. Following oral dosing in humans, curcumin can be detected as a water-soluble glucuronide and sulfate conjugate in plasma. In rats and humans, curcumin undergoes sulfation catalyzed by human phenol sulfotransferase 1A1 (SULT1A1) and human phenol sulfotransferase 1A3 (SULT1A3), while the glucuronidation of curcumin is catalyzed by uridine diphosphate-glucuronosyltransferase (UGT) [17]. When curcumin is reduced or conjugated, it forms species that are less effective at inhibiting the expression of cyclooxygenase-2 (COX-2) than curcumin itself. Curcumin sulfate, hexahydrocurcumin, and tetrahydrocurcumin all inhibit prostaglandin E2 to varying degrees, whereas hexahydrocurcuminol shows no activity at all [13]. Metabolites of curcumin other than tetrahydrocurcumin have much lower biological activity compared to curcumin itself [18,19]. Various strategies, including piperine to block glucuronidation, curcumin nanoparticles, curcumin phospholipid complexes, structural curcumin analogues, and curcumin in liposomes, are employed to enhance curcumin’s bioavailability [2,13].

## 3. Antimicrobial Properties of Curcumin

### 3.1. Antiviral Effect of Curcumin

**Figure 2:**
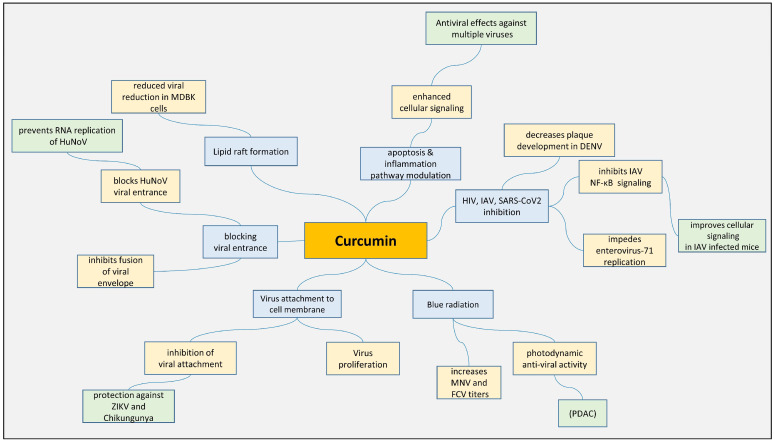
The antiviral mechanisms of curcumin and its pharmacological effects.

Curcumin exhibits effectiveness against numerous viruses, including those responsible for feline infectious peritonitis, respiratory syncytial virus (RSV), parainfluenza type 3, herpes simplex virus (HSV), vesicular stomatitis, and flock house [20]. Combating viral infections, particularly those caused by constantly mutating viruses, has long been a challenge. Curcumin can modulate various molecular targets involved in cellular signaling pathways, including apoptosis and inflammation, through intermolecular interactions (Figure 2), thereby exerting its antiviral effects [21].

The initial step in viral infection involves the attachment of the virus to the cell membrane surface, enabling its proliferation. Recent research suggests that curcumin can inhibit the attachment of arthropod-borne viruses to cell surfaces, offering protection against Zika virus (ZIKV) and Chikungunya infections [22]. In a dose-dependent manner, curcumin reduced the overall viral production in Madin-Darby bovine kidney (MDBK) cells by enhancing lipid raft formation, thereby affecting the entry stage of bovine herpesvirus type 1 [23]. Furthermore, curcumin consistently prevented the entry of various genotypes of the hepatitis C virus by altering viral envelope fluidity, thus impeding viral fusion [24].

Curcumin also inhibits the early stages of infection by non-enveloped viruses, such as human norovirus (HuNoV). Its antiviral effects, such as blocking viral entry rather than HuNoV RNA replication, are dose and time dependent [25]. Since the HuNoV virus does not target the lipid bilayer envelope structure, curcumin influences viral entry by inhibiting the activity of virus surface proteins [25].

Additionally, the efficacy of employing photodynamic treatment with 5 µM concentrations of curcumin has been investigated. This method involves exposing the chemical to specific light wavelengths, activating it and leading to the production of reactive oxygen species. Photodynamic triggering by blue light radiation demonstrates stronger effects on murine norovirus (MNV) titers compared to curcumin or blue light alone [26].

Further research evaluated the efficacy of photodynamic anti-norovirus (PDAC) treatment against feline calicivirus (FCV), HuNoV, and MNV. While treatment with curcumin at concentrations up to 13.6 M was more effective against FCV than MNV, it reduced the 50% tissue culture infective dose (TCID50) of both viruses [27]. Employing PDAC against noroviruses in oysters presents a potentially simple method to prevent norovirus contamination, particularly as curcumin is now considered safe for use in the food industry.

Curcumin has demonstrated antiviral activity against ZIKV by blocking the virus’s ability to adhere to cells. Its main action is inhibiting ZIKV entry and attachment rather than later stages of infection, as evidenced by its ability to reduce ZIKV recovery in cells treated before or after infection in multiple time-of-addition experiments [22]. A study was conducted to assess curcumin’s anti-ZIKV activity, revealing an IC50 value against multiple ZIKV strains in plaque experiments conducted in Vero E6 cells. A time-of-addition analysis established curcumin’s interference with ZIKV cell attachment [28]. These findings suggest curcumin’s potential to inhibit ZIKV.

Similarly, incubating vesicular stomatitis virus with curcumin at a dosage of 4.5 µM prevented viral infection [29]. Furthermore, curcumin effectively decreased viral production and absorption when incubated with enveloped viruses causing transmissible gastroenteritis at a dosage of 20 µM [30]. Incubation with curcumin at a dosage of 15 µM reduced infection by porcine reproductive and respiratory syndrome virus, inhibiting membrane fusion and internalization processes and increasing cell survival while inhibiting viral proliferation in viral hemorrhagic septicemia viruses [31].

Most research on curcumin’s antiviral benefits has focused on its efficacy against human immunodeficiency virus (HIV). Curcumin affects various stages of the HIV life cycle, hindering viral integration through direct interactions with the protein catalytic core. A synthetic curcumin analogue, curcumin A, evaluated for anti-HIV-1 activities, exhibited stability in tissue culture media, phosphate buffer, and serum when tested in vitro [32]. Tetrahydrocurcumin, a colorless curcumin metabolite, showed promise as a topical vaginal microbicide for HIV prevention, suppressing HIV-1 more efficiently than medication alone in cell lines [33].

Curcumin’s effectiveness against dengue virus (DENV) was investigated alongside its anti-HIV activity. Curcumin decreased plaque development with minimal harm in four DENV strains tested, acting at the cellular level rather than targeting viral functions [34,35]. Curcumin and its synthetic analogues modestly inhibited viral protease activity in vitro, while curcumin impeded Chikungunya virus (CHIKV) entry, preventing infection [29,35,36].

Curcumin is one of the most potent inhibitors of influenza A viruses (IAVs), acting at multiple stages of the virus life cycle. It disrupts the haemagglutinin activity of the virus, reducing infectivity when incubated with IAV. Curcumin also inhibits the NF-κB signaling system, thus showing antiviral potential against IAV [37,38,39]. In a lung tissue model, curcumin-treated mice showed improved survival and reduced weight loss after IAV infection [40]. Partial inhibition of IAV activity is attributed to the enone functional groups in curcumin, which form conjugates with viral surface proteins, interfering with viral function [41]. Curcumin effectively decreased mRNA levels in the M gene of infected IAV cells at the highest safe doses [42].

Curcumin targeted Vero cells, inhibiting enterovirus-71 replication and significantly reducing genome replication and protein expression in intestinal epithelial cells [43,44]. Speculation surrounds curcumin’s potential to limit SARS-CoV-2 replication given its antiviral effects on SARS-CoV-1. Curcumin inhibits the cytokine storm associated with viral infections by preventing the synthesis of proinflammatory cytokines [45]. Molecular docking investigations reveal curcumin’s potential to inhibit SARS-CoV-2 replication by interacting with the spike glycoprotein and blocking primary viral protease, viral nonstructural proteins, and angiotensin-converting enzyme-2 [46,47].

### 3.2. Antibacterial Effect of Curcumin

Curcumin inhibits the growth of both Gram-negative and Gram-positive bacteria [48,49].

Curcumin exhibited minimum inhibitory doses (MICs) ranging from 125 to 250 g/mL against 10 distinct strains of methicillin-resistant *Staphylococcus aureus* (MRSA). It should be emphasized that curcumin alone exhibited an MIC of 250 g/mL against MRSA. However, when curcumin was combined with an antibiotic, the MIC was considerably reduced to 125 g/mL. In the crossed-out test, the MICs of the anti-MRSA medicines oxacillin (OXI), ampicillin (AMP), ciprofloxacin (CIP), and norfloxacin (NOR) were considerably reduced in the presence of curcumin. Curcumin was discovered to decrease the MICs of several antibiotics that were examined, including OXI, AMP, CIP, and NOR. These study findings suggest that new combinations of antibiotics containing curcumin could be created to fight against MRSA infections [50]. Furthermore, in vitro studies conducted on individuals with gastrointestinal diseases demonstrated curcumin’s ability to reduce the growth of all species of *Helicobacter pylori* (*H. pylori*) [51].

**Figure 3:**
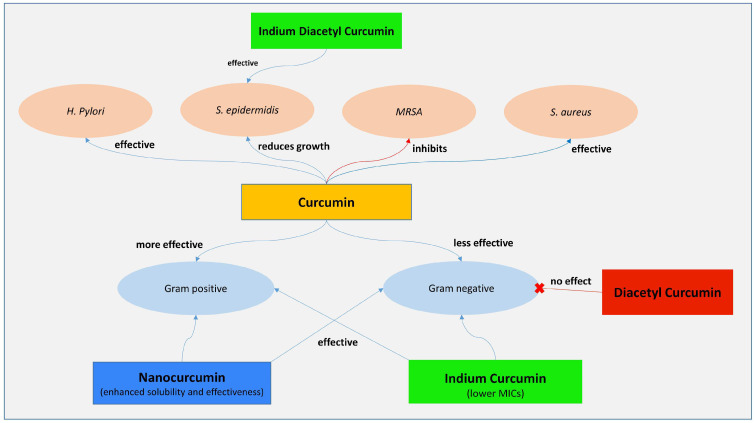
The antibacterial mechanisms of curcumin and its pharmacological effects.

In a research, the antimicrobial action of curcumin was evaluated using the pour plate method. Various curcumin fractions were tested for antimicrobial susceptibility against *Pseudomonas aeruginosa*, *Staphylococcus aureus*, *Bacillus subtilis*, *Bacillus cereus*, *Escherichia coli*, and *Bacillus coagulans*. Curcumin exhibited strong antibacterial effects against all bacterial strains tested (Figure 3) [52]. Researchers compared curcumin to three new compounds—indium curcumin, diacetyl-curcumin, and indium diacetyl-curcumin—in vitro to assess their antibacterial effects. According to their findings, curcumin has antibacterial properties against all of the species tested. Indium diacetyl-curcumin was found to have an effect against *Staphylococcus epidermidis* and *Staphylococcus aureus*, while diacetyl-curcumin did not exert any antibacterial effects on any of the species tested. Indium curcumin, on the other hand, showed strong antibacterial potential against every single strain tested. Curcumin has an MIC of 187.5 µg/mL against *Staphylococcus aureus* and 46.9 µg/mL against *Staphylococcus epidermidis*, according to their determination of the MICs for all substances tested against these bacteria. The MIC for indium curcumin was found to be slightly lower than that of the two species mentioned earlier, with MICs of 93.8 µg/mL and 23.4 µg/mL, respectively. According to their findings, indium curcumin is more effective against bacteria than curcumin [53].

A spectrofluorophotometric analysis was employed to test the antimicrobial activity of various curcumin concentrations against periodontopathic microbes, including *Streptococcus gordonii*, *Prevotella intermedia*, *Porphyromonas gingivalis*, *Treponema denticola*, *Fusobacterium nucleatum*, and *Aggregatibacter actinomycetemcomitans*. Curcumin inhibited the growth of *T. denticola*, *P. intermedia*, *P. gingivalis*, and *F. nucleatum* at various doses [54]. Another study demonstrated curcumin’s ability to inhibit the growth of several bacteria, including *Enterococcus faecalis*, *Prevotella intermedia*, and *P. gingivalis*, and other periodontal microbes like *Lactobacillus casei*, *Streptococcus mutans*, and *Actinomyces viscosus*, except for *E. coli* [55].

The antibacterial capabilities of curcumin nanoparticles against various bacteria, including *Aspergillus niger*, *Bacillus subtilis*, *E. coli*, *S. aureus*, *Pseudomonas aeruginosa*, and *Penicillium notatum*, were investigated [56]. Curcumin nanoparticles ranging from 2 to 40 nm in size, produced via the wet milling process, were soluble in freshwater without surfactants. Nanocurcumin exhibited enhanced water solubility and antibacterial properties, particularly effective against Gram-positive bacteria [56]. Core–shell copper oxide–curcumin nanocomposite technology was developed to combat *Shigella dysenteriae*, *Streptococcus pneumoniae*, *E. coli*, and *S. aureus*, demonstrating superior antibacterial activity compared to amoxicillin [57,58].

Additionally, isolated curcumin bioactive compounds, including curcumin, bisdemethoxycurcumin, and demethoxycurcumin from *Curcuma longa*, were tested for their anti-Clostridium activity. Curcumin significantly inhibited the growth of *Clostridium difficile* cultures at doses ranging from 6 to 33 μg/mL, with no impact on common bacteria found in the human digestive tract [59]. Figure 3 illustrates key antibacterial pathways influenced by curcumin.

Multiple in vitro studies have demonstrated curcumin’s ability to inhibit the growth of various microorganisms, including both Gram-positive and Gram-negative bacteria [39]. These antibacterial activities are attributed to curcumin’s extensive network of molecular mechanisms, including inhibition of DNA replication, changes in plasmid gene expression, degradation of cell membranes, and reductions in motility. Researchers investigated curcumin’s antibacterial properties using an animal model, focusing on its impact on mice infected with *H. pylori* and its potential to reduce stomach damage caused by infection, as assessed through histopathology. The study revealed that curcumin effectively eliminated *H. pylori* from animals and aided in repairing stomach injuries induced by the bacterium, suggesting significant preventive benefits against *H. pylori* infections [39,51].

Additionally, the antibacterial activity of curcumin was evaluated in a mouse model [60]. The study demonstrated that animals treated with curcumin were protected against strains of *S. aureus*-induced pneumonia, including methicillin-resistant *S. aureus* (MRSA). Curcumin was found to block a novel pathway, preventing α-hemolysin from generating pores, which are crucial for the development and spread of *S. aureus* pneumonia. This discovery paves the way for the development of improved antimicrobial medications to combat *S. aureus* infections. Furthermore, supplementation with curcumin significantly reduced the injury caused by α-hemolysin to alveolocytes when co-cultured with *S. aureus* [60]. In vivo testing of nanoparticles containing Nisin loaded with curcumin demonstrated their efficacy in combating wound infections. Curcumin exhibited promising antibacterial properties in burn wound infections, as evidenced by a considerable decrease in wound size observed in mice [61]. Similarly, curcumin showed potential as an antibacterial agent in an in vivo study involving *E. coli* isolates resistant to multiple drugs [62]. Table 1 provides a summary of the experimental research conducted to assess curcumin’s antibacterial properties.

Table 2 presents a summary of experimental studies aimed at identifying curcumin’s antiviral effects. In the summary of experiments below, the researchers maintained the concentration of curcumin at the lowest physiological level.

## 4. Nanoformulations Based on Curcumin

**Figure 4:**
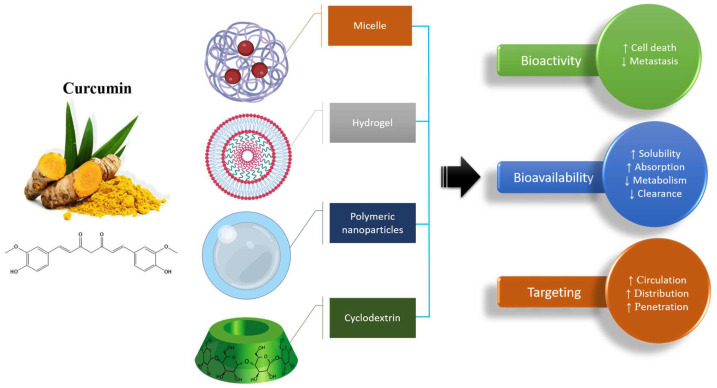
Nanoformulations of curcumin and their advantages in practical applications; (↑ increase, ↓ decrease).

There is a plethora of curcumin formulations that incorporate volatile oil, piperine, and lecithin [79,80,81,82]. Orally administered, these compounds surpass the absorption capacity of pure curcumin. Recent innovations have yielded diverse curcumin formulations such as liposomes, micelles, phospholipid complexes, cyclodextrins, nanoparticles, emulsions, and hydrogels, among others. These formulations augment the effectiveness of curcumin by enhancing circulation, improving absorption, increasing resistance to metabolic processes, promoting enhanced absorption in the small intestine, and lengthening plasma half-life (Figure 4) [83,84,85]. Research on healthy individuals compared standardized curcumin mixtures (CSs) to phytosome formulations containing curcumin, volatile oils of turmeric rhizome, or a combination of hydrophilic carriers, cellulose derivatives, and natural antioxidants (CHC). When compared to regular curcumin, the CHC formulation considerably raises blood curcuminoid levels [80]. The molecular inclusion complexes that cyclodextrins (CDs) can create with lipophilic drugs improve the active components’ water solubility, dispersion, and absorption. The study examined how well the curcumin formulation with γ-cyclodextrin was absorbed into the bloodstream. Two commercially available formulations—one containing curcumin phytosomes (CSLs) and the other containing curcumin with turmeric essential oils (CEO)—were compared to this one, as was a standardized curcumin extract. When incorporated in a formulation with γ-cyclodextrin, curcuminoids exhibit enhanced absorption in healthy individuals [86]. The coprecipitation approach was used to create the curcumin–β-cyclodextrin inclusion complex. For curcumin, the formation of an inclusion complex improved its solubility in water from 0.00122 to 0.721 mg/mL. Under simulated gastric circumstances, researchers examined how conventional poly(N-isopropylacrylamide/sodium alginate) hydrogels cross-linked with nanoclay released the inclusion complex, while nanocomposite hydrogels released N,N′-methylenebisacrylamide (BIS). The release–swelling ratio was lowest for hydrogels at pH = 1.2 and highest at pH = 6.8, correspondingly. Nanocomposite hydrogels exhibited a decreasing swelling coefficient and cumulative release as the nanoclay concentration increased. On the other hand, traditional hydrogels showed an inverse relationship between the BIS ratio and swelling ratio–cumulative release [87]. In a study, researchers employed the polyol dilution method to generate curcumin liposomes. The lipid phase consisted of a 9:1 molar ratio of hydrogenated phosphatidylcholine to cholesterol and glycerin, propylene glycol, and polyethylene glycol 400 were used to dissolve the polyols [88]. The curcumin content and liposome size are both influenced by the polyol type and concentration. When making liposomes, the preparation temperature is crucial as well [88]. Using liposomes containing varying amounts of hydrogenated phospholipids, researchers investigated curcumin’s stability and release performance [89].

An alternate vehicle for human medication delivery could be liposomes coated with chitosan. In one study, the authors assessed the practicality of anionic and chitosan-coated liposomes containing curcumin. By assessing the bioavailability of ingested curcumin, the formulations’ application was assessed in vitro. Researchers have found that curcumin is more bioavailable when it is bound to a liposome with a positively charged surface since this surface improves absorption in the small intestine [90]. To enhance the solubility and absorption of curcumin following topical application to the skin, a nanoemulgel was created from a curcumin nanoemulsion that had been originally developed as a low-energy emulsion. The gelling agent used in this conversion was cross-linked polyacrylic acid (Carbopol^®^ 934). When compared to curcumin and betamethasone-17-valerate gel, the nanoemulgel formulation accelerated wound healing in psoriatic mice. The curcumin nanoemulgel formulation shows promise as a long-term effective treatment for psoriasis, according to the studies [91].

Researchers have recently discovered that curcumin can protect the stomach lining from a variety of harmful substances, including NSAIDs, stress hemorrhage, and (*H. pylori*) infection, as well as against gastric mucosal injury in rats. Through modulating associated signal pathways, curcumin exhibits substantial anti-inflammatory and anti-cancer effects via DNA methylation and histone modification [92]. Recurrence of tumors following surgery and metastasis can be effectively prevented using curcumin nanoemulsions [93]. An eyedrop formulation that contains thermosensitive hydrogel, latanoprost, and curcumin nanoparticles for dual drug delivery was developed. The dual drug delivery system that was created has demonstrated many positive characteristics, including a longer release profile, biocompatibility in both laboratory and living organism settings, decreased cell inflammation and apoptosis, and protection of Trabecular Meshwork (TM) cells from oxidative damage [94]. Patients hospitalized with COVID-19 can recuperate much more quickly when given an oral dosage of nanocurcumin [95]. Inhibiting the spread of breast and lung cancer was achieved by the use of the hybrid curcumin–phospholipid complex, which was administered orally [96]. To enhance curcumin’s oral bioavailability, solubility, and flow the researchers created a high-performance formulation of the phospholipid complex [97]. Curcumin can be released gradually into the bloodstream by polymer micelles that are created using the block copolymer methoxy-poly(ethylene glycol)-poly(caprolactone) (PCL) [98].

The effectiveness of doxorubicin and curcumin delivered simultaneously using peptide hydrogel in treating cells of head and neck cancer was investigated in the research [99]. A double-peptide hydrogel containing medicines was found to be therapeutically useful for the local treatment of head and neck cancer, according to the findings [99]. LRA-CS, a hydrogel composed of amylopectin and chitosan, was developed and evaluated for curcumin delivery. The authors studied the release properties of curcumin in capsules in the simulated stomach and intestinal liquids. The results demonstrated that curcumin remained stable in the stomach and was subsequently released into the small intestine through the LRA-CS hydrogel [100]. Another option for curcumin distribution was a chitosan–nanocellulose hydrogel modified with a nonionic surfactant [101]. Curcumin and resveratrol were transdermally co-delivered using a hydrogel based on cyclodextrin nanospigoid (CDNS). Compared to conventional curcumin and resveratrol, nanosponges increased the in vitro release of curcumin by a factor of ten and resveratrol by a factor of two and a half. The cytotoxic effect on MCF-7 cells was found to be synergistic when curcumin–CDNS and resveratrol–CDNS were combined. Curcumin–CDNS and resveratrol–CDNS into a hydrogel foundation was created using carbomer and propylene glycol. When compared to the hydrogel created without CDNS, the photostability of curcumin was nearly five times higher and that of resveratrol was seven times higher in the CDNS hydrogel formulation. The use of a CDNS–hydrogel base to provide curcumin and resveratrol greatly increases its absorption [102]. The hydrogel system of oxidized cellulose and polyvinyl alcohol was frozen to include curcumin. This approach has been shown to facilitate natural wound healing in rats through in vitro investigations [103]. Another group created advanced ultrasol curcumin (AUC), a novel curcumin formulation with enhanced intestinal stability, excellent bioavailability and when given at the recommended levels, AUC ameliorates the pathophysiology of rat osteoarthritis [104].

On healthy volunteers, researchers compared the oral bioavailability of three different curcumin formulations: Curene^®^ is a bioavailable formulation of Curcucma longa extract comprising naturally derived curcuminoids formulated with proprietary Aquasome^®^ technology. Curene^®^, which was recently developed, CP-01, which contained volatile turmeric oil, and 95% conventional curcuminoids were given to the volunteers. Trial participants who were otherwise healthy were able to safely take Curene^®^, and its oral bioavailability was determined to be 95%, far higher than that of CP-01 and conventional curcuminoids [105]. At two months of age, both wild-type and GFAP-IL6 mice were tested to determine Longvida^®^ Optimized Curcumin’s (LC) anti-inflammatory activity. In GFAP-IL6 mice, LC reduces inflammation, which in turn reduces neurodegeneration and motoric abnormalities [106]. Various commercial curcumin formulations are available, each with its own set of specifications for bioavailability and pharmacokinetics. These include micronized curcumin, NovaSol (micellar curcumin), CurcuWin, Biocurcumax (Curcumin C3 Complex^®^+Bioperine), Cavacurmin, and Theracurmin. Commercial formulations with bioavailability more than 100 times that of reference curcumin stand out; these include NovaSol^®^ (185 mg), Curcuwin^®^ (136 mg), and LongVida^®^ (100 mg) [107].

The antibacterial potential of nanofibers made of curcumin-loaded silica nanoparticles was tested against MRSA. The antibacterial effects were more pronounced than those of pure curcumin, according to both in vitro and in vivo studies [108,109]. To test the antibacterial properties of curcumin-loaded polycaprolactone-gum tragacanth nanofibers, a research group developed them. Nanofibers coated with curcumin demonstrated a high level of effectiveness against S. aureus, with a reduction of 99.9 percent [110]. Nanofibers coated with curcumin and polyurethane demonstrated curcumin’s synergistic antibacterial activity [111]. Nanofibers with potent bactericidal properties were developed by incorporating a mix of polymers loaded with curcumin [112]. Curcumin exhibited improved water diffusion when delivered by nanocrystals ranging in size from 2 to 40 nm. Because of their small particle size, the curcumin-loaded nanocrystals had a high bioavailability, which led to their strong antibacterial effects [113]. The increasing bacterial drug resistance to antibiotics poses a significant threat to public health, leading to serious infections and deaths without effective therapies. A dynamic covalent polymeric antimicrobial, based on phenylboronic acid (PBA)-installed micellar nanocarriers containing clinical vancomycin and curcumin, has been developed to overcome drug-resistant infections. The antimicrobial’s formation is facilitated by reversible dynamic covalent interactions between PBA moieties and vancomycin diols, providing stability in blood circulation and excellent acid responsiveness in the infection microenvironment. This combination therapy shows biocompatibility without unwanted toxicity, making it a universal platform to combat drug-resistant infectious diseases [114].

Another study presents an intelligent nanoparticle system (CCM+TTD@ZIF-8 NPs) that targets bacteria, promotes fluorescence imaging, and uses pH-response-guided photodynamic therapy (PDT) for infection-induced wound healing. The CCM+TTD@ZIF-8 NPs have multiple functions, including targeting drug-resistant bacteria, promoting specific ROS accumulation, and showing excellent bactericidal efficacy against *Staphylococcus aureus* and *Pseudomonas aeruginosa* strains. The system also accelerates the healing process of infected burn wounds through the regeneration of epidermal tissue, angiogenesis, and collagen deposition. This multifunctional system has great potential for infected burn wound healing [115].

## 5. Combination Therapy Using Curcumin

The alleged therapeutic benefits of natural materials are enhanced in combination therapy. The oral administration of nanoparticles as therapeutic agents, on the other hand, has the potential to circumvent some of selective chemotherapy’s downsides while simultaneously providing advantages, such as reduced toxicity and the promotion of antibiotic resistance [116,117,118]. The resistance problem can also be addressed by the use of combination medicines. The antibacterial activity of curcumin was enhanced when it was hybridized with octa-arginine. The outcome was a synergistic impact that killed bacteria by preventing curcumin from reaching their cell membranes [119]. The same is true of bacteriocins, another short peptide that has shown effective antibacterial action but is unable to produce the intended therapeutic effect because of resistance. To combat Staphylococcus epidermidis and Escherichia coli, curcumin was given alongside bacteriocins. The antibacterial activity of bacteriocins was enhanced by curcumin, suggesting that the two compounds have biological uses when combined [120]. The water solubility and potency of curcumin were enhanced by co-delivering it with suberoylanilide hydroxamic acid. The results demonstrated that the antibacterial effect of curcumin was enhanced by this synergistic combination compared to suberoylanilide hydroxamic acid and pure curcumin alone [121]. The majority of deaths caused by infections are caused by drug-resistant varieties of Staphylococcus aureus. The emergence of drug resistance has rendered many antibiotics ineffective. Because antibiotics have poor penetration and retention in mammalian cells, latent infections can recur. The limited penetration and hydrophobicity of curcumin and berberine made them less effective when used alone. Again, there was no discernible synergistic impact on microbes when the two were administered together. Nonetheless, there was a notable synergistic impact against MRSA when both were loaded into liposomes [122]. The authors looked at the antibacterial effects of curcumin with xylitol. Synergistic effects against Staphylococcus aureus, Pseudomonas aeruginosa, and Candida albicans were seen as a result of the combined effect [123]. Synergy between curcumin and polymyxin-B was demonstrated in a recent in vitro investigation against both Gram-positive and Gram-negative bacteria [124].

## 6. Limited Curcumin-Based Clinical Trials

On the website “National Institutes of Health Clinical Trial.gov”, a search for the term “curcumin” returned fewer than 300 clinical studies. Fifty percent of these studies were found complete and pertained specifically to the application of curcumin in the context of cancer and other chronic diseases. Unfortunately, the number of clinical trials investigating antimicrobial activity was found to be quite limited. The essential particulars of some antimicrobial clinical trials are presented in Table 3. It is evident from the available data in the table that curcumin has continued to be the subject of scientific investigation for the last twenty years. Due to several factors, its clinical trials have not produced satisfactory results. The aforementioned drawbacks encompass a limited number of study endpoints (e.g., safety, tolerance, and form), insufficiently regulated variations in curcumin interventions (e.g., dosage, form, and timing), and the application of small sample sizes to specific disease categories. It is worth noting that the public accessibility of these clinical studies is considerably restricted. This suggests that curcumin still faces a substantial obstacle in its journey toward practical clinical applications [125].

## 7. Side Effects Associated with Curcumin

People seem to place great trust in the safety of curcumin due to centuries of experience with its consumption in the diet. An increasing number of individuals are turning to dietary supplements derived from natural plant sources, with curcumin being a notable example [133]. In an in vivo study, the oral administration of 3500 mg/kg of curcumin showed no adverse effects [134]. A clinical trial involving patients with rheumatoid arthritis who orally consumed varying dosages of 1200–2100 mg of curcumin daily for six weeks reported no side effects [135]. Additionally, intervention with curcumin in advanced colon cancer patients demonstrated generally good tolerability at doses of up to 3600 mg daily for four months, although some patients experienced nausea and diarrhea, which subsided spontaneously in some cases [136,137]. The European Union Food Science Committee has established a daily intake recommendation of 0–3 mg/kg of curcumin [133]. While most clinicians agree that dietary curcumin poses very low toxicity and does not pose significant risks to human health, it does not mean that it is entirely risk free. Side effects from excessive curcumin intake remain a possibility. Therefore, it is crucial to exercise caution when considering the excessive use of curcumin in any of its forms.

## 8. Conclusions and Future Perspective

Curcumin’s robust antimicrobial properties make it a versatile agent capable of targeting a wide range of bacteria. Its effectiveness against pathogens like *H. pylori*, both independently and in conjunction with traditional antibiotics, underscores its potential as a broad-spectrum antibacterial agent. While in vitro and in vivo studies support its efficacy, the lack of clinical trials evaluating curcumin’s direct antibacterial activity hinders its translation into clinical practice.

Despite its promising attributes, curcumin poses challenges in terms of bioavailability, toxicity, and solubility, whether administered orally or intravenously. Nevertheless, its demonstrated antiviral capabilities and effectiveness in combating food-borne microorganisms through synergistic combination therapies highlight its therapeutic potential. Overcoming physiological barriers, such as hydrophobicity and poor bioavailability, is crucial to maximizing curcumin’s therapeutic effectiveness.

Nanoformulations of curcumin offer a promising avenue to address these challenges and enhance its bioactivity. Recent advancements in nanotechnology have spurred interest in developing curcumin-based nanoformulations with improved antimicrobial properties. While research indicates promising bioactivity against drug-resistant infections, further studies are needed to assess the safety and environmental impact of curcumin nanoparticles.

In conclusion, curcumin stands as a promising candidate for next-generation antimicrobial therapy, especially when integrated into nanoformulations. By addressing key factors such as bioavailability and safety, curcumin has the potential to emerge as an effective solution in combating antibiotic-resistant bacteria and advancing antimicrobial therapy. Continued research and development efforts are essential to harnessing the full therapeutic potential of curcumin and its nanoformulations.

## Figures and Tables

**Table 1:** A review of the research on curcumin’s antibacterial activity.

Type of Bacteria	Experiment	Minimum Inhibitory Concentration (µg/mL)	Outcome	Reference
*S. aureus*,	In vitro	25	Curcumin ruptured cell membranes and inhibited all tested microorganisms, showing significant antibacterial action	[63]
*E. coli*,				
*Enterococcus faecalis*, and *Pseudomonas aeruginosa*				
*E. coli*	In vitro	12	Curcumin strongly inhibited *E. coli*	[64]
*Klebsiella pneumonia*,	In vitro	34	When compared to demethoxycurcumin and bisdemethoxycurcumin, curcumin exhibited far stronger antibacterial action	[65]
*Bacillus subtilis*,				
*Enterobacter aerogenes*,				
*E. coli, S. aureus*,				
*Proteus mirabilis*, and				
*P. aeruginosa*				
*E. coli*	In vitro	8	Curcumin inhibited the levofloxacin-induced SOS reaction in *E. coli*	[66]
*P. aeruginosa*	In vitro	8–512	Antibacterial synergy was seen when curcumin, azithromycin, and gentamicin were combined	[67]
*S. aureus*	In vivo and in vitro	2–16	Curcumin healed *S. aureus*-infected mice	[60,68]
*Salmonella typhimurium* and	In vivo and in vitro	0.5–2	The mouse model exhibited the strong antibacterial action of curcumin	[69]
*Salmonella typhi*				
*H. pylori*	In vivo and in vitro	5–50	In mice, curcumin eradicated *H. pylori* that caused stomach damage	[70]

**Table 2:** A review of the research on curcumin’s antiviral activity.

Type of Virus	Curcumin	Outcome	Reference
Hepatitis B virus	10 μM	Changes in the effects of curcumin on hepatitis B virus entrance	[71]
Chikungunya virus and Zika virus	5 μM	The antiviral effects of curcumin on the chikungunya and Zika viruses	[22,72]
Human immunodeficiency virus 1 (HIV-1)	20 nM	Immunomodulatory effects of HIV-1 on silver nanoparticles stabilized with curcumin	[73]
Enterovirus 71 (EV71)	40 μM	The activity of curcumin against EV71 virus	[43,74]
Kaposi’s sarcoma-associated herpesvirus (KSHV or HHV8)	20 μM	The antiviral effects of curcumin on the replication and development of KSHV	[75]
Zika virus	25 μM	Zika virus inhibitory effects were observed in the activity	[76]
Dengue virus	40 μM	Curcumin has antiviral efficacy against several serotypes of dengue virus	[30,77]
Human parainfluenza virus type 3	10–30 μM	Inhibitory effects of curcumin on the replication of the human parainfluenza virus	[78]

**Table 3:** Curcumin clinical studies for the management of microbial diseases.

Disease	Number of Participants	Dose	Result	Reference
Chronic periodontitis	23	1% curcumin solution, 0.2% chlorhexidine gluconate, and saline were used as irrigants	The curcumin group reduced bleeding scores more effectively than the control group	[125]
Oral plaque	27	LED lighting generating blue light with a curcumin concentration of 30 mg/L	Curcumin possesses the capability to disintegrate mouth plaque; salivary microbes can be reduced using the blue LED device that contains curcumin	[126]
Periodontitis	25	Locally applied 1% curcumin gel to treat periodontitis	A curcumin gel application dramatically lowered the periopathogen microbiological count; when used after scaling and root planing, curcumin gel effectively inhibits the growth of oral germs	[127]
Peptic ulcer	30	Curcumin (500 mg) along with piperine (5 mg)	Dyspepsia symptoms improved	[128]
Peptic ulcer	21	Clarithromycin (500 mg) + amoxicillin (1 g) + esomeperazole (20 mg) + curcumin (500 mg)	Reduction in pro-inflammatory IL-1B level; improved healing	[129]
Chronic gastritis	24	Omeprazole (20 mg) + amoxicillin (1 g) + metronidazole (800 mg) + curcumin (700 mg)	Reduction in oxidative DNA damage; improved clinical symptoms	[130]
COVID-19	70 mild to severe COVID-19 patients	Patients took a dietary supplement containing 2.5 milligrams of bioperine and 252 milligrams of curcumin	Reduced the length of hospital stays for patients with moderate to severe symptoms, showed early symptomatic recovery	[131]
COVID-19	30 mild to moderate COVID-19 patients	Patients in the Sinacurcumin^®^ soft gel 40 mg group took two gels with meals: breakfast and dinner	Symptoms such as chills, cough, and smell/taste disturbances cleared up significantly faster than in the control group, with the exception of sore throat	[132]
COVID-19	115	Dietary supplement: palmitoylethanolamideDietary supplement: curcumin	Curcumin supplementation reduced the proinflammatory response in patients recently diagnosed with COVID-19	[76]

## Data Availability

Not applicable.
